# Old men with prostate cancer have higher risk of Gleason score upgrading and pathological upstaging after initial diagnosis: a systematic review and meta-analysis

**DOI:** 10.1186/s12957-021-02127-3

**Published:** 2021-01-20

**Authors:** Xiaochuan Wang, Yu Zhang, Zhengguo Ji, Peiqian Yang, Ye Tian

**Affiliations:** Department of Urology, Capital Medical University affiliated Beijing Friendship Hospital, No. 95, Yongan Road, Xicheng District, 100050 Beijing, People’s Republic of China

**Keywords:** Systematic review, Meta-analysis, Prostate cancer, Gleason grading system, Neoplasm staging, Age

## Abstract

**Background:**

To evaluate the predictive performance of age for the risk of Gleason score change and pathologic upstaging.

**Evidence acquisition:**

Ovid MEDLINE, Ovid Embase, and the Cochrane Library were searched from inception until May 2020. Quality of included studies was appraised utilizing the Newcastle-Ottawa Quality Assessment Scale for case-control studies. The publication bias was evaluated by funnel plots and Egger’s tests.

**Evidence synthesis:**

Our search yielded 27 studies with moderate-to-high quality including 84296 patients with mean age of 62.1 years. From biopsy to prostatectomy, upgrading and upstaging occurred in 32.3% and 9.8% of patients, respectively. Upgrading from diagnostic biopsy to confirmatory biopsy was found in 16.8%. Older age was associated with a significant increased risk of upgrading (OR 1.04, 95% CI 1.03–1.05), and similar direction of effect was found in studies focused on upgrading from diagnostic biopsy to confirmatory biopsy (OR 1.06, 95% CI 1.04–1.08). For pathologic upstaging within older men compared with younger, the pooled odds was 1.03 (95% CI 1.01–1.04).

**Conclusion:**

Thorough consideration of age in the context of effect sizes for other factors not only prompts more accurate risk stratification but also helps providers to select optimal therapies for patients with prostate cancer.

**Supplementary Information:**

The online version contains supplementary material available at 10.1186/s12957-021-02127-3.

## Background

Increasing prostate cancer (PCa) morbidity and mortality over past decades prompted the screening for general population and early detection for high-risk groups, as well as the improvement of manifold treatment modalities. PCa represents a spectrum of diseases that range from aggressive diseases that may require treatment to non-aggressive diseases that may do not. It is universally acknowledged that overdiagnosis and overtreatment could be averted by rational use of active surveillance (AS) and observation. Within younger informed men with long expectancy of life and favorable health status, patients diagnosed with low-to-intermediate-risk PCa may be considered for AS [1]. Compared to immediate curative treatment, AS delays the onset of potential side effects and is not associated with increased PCa mortality [2]. As for older patients with short expectancy of life and unfavorable health status, observation is recommended in order to maintain the quality of life and avoid the adverse events of unnecessary therapies [1]. Within this patient group, the survival rate of deferred treatment was also similar to that of immediate treatment [3]. Therefore, age is a pivotal factor for PCa in both risk appraisal and decision-making of treatment.

At initial diagnosis, clinical stage and Gleason score (GS) are both critical factors which provide urologists with the clinical profile of PCa to discriminate between indolent and aggressive disease. However, as diagnostic tools, both imaging tests and biopsy examinations inevitably have limitations in reflecting the tumor nature. Even if Gleason grading system has been modified over time, the accuracy of biopsy GS for predicting prostatectomy GS was reported to be barely satisfactory (53–74%) [4]. Pathological upstaging (hereafter referred to as upstaging) to more aggressive diseases was also reported to affect 7.2–17.2% of individuals [5, 6]. It has been demonstrated in large-scale studies that GS upgrading (hereafter referred to as upgrading) and upstaging were significantly associated with biochemical recurrence, distant metastasis and death from PCa [7, 8], while GS downgrading (hereafter referred to as downgrading) was a protective factor [9]. Various predictors such as prostate-specific antigen (PSA), prostate volume (PV), and number of biopsy cores were found to be associated with upgrading, downgrading or upstaging [10]. Age was also found to be predictive; however, value of odds ratio was slightly greater or less in some studies [11, 12]. Furthermore, most studies were based on monocentric databases and limited population. To address these matters, we aimed to conduct a comprehensive review of literatures and investigate the correlation between age and the risk of GS change (upgrading or downgrading) and upstaging in this systematic review and meta-analysis.

### Evidence acquisition

Our review was preformed according to the Preferred Reporting Items for Systematic Reviews and Meta-analysis (PRISMA) guideline [13]. Methods and criteria were specified in advance and documented in a protocol as a reference for our investigators.

#### Search strategy

A comprehensive literature search for eligible records was conducted via the following databases from inception until 5th May 2020: Ovid MEDLINE, Ovid Embase, and the Cochrane Library. The full PubMed search strategy was shown in Additional Figure 1. Search terms combined MeSH major topic with free-words. The major terms included ‘Prostatic Neoplasms,’ ‘Multivariate Analysis,’ ‘Odds Ratio,’ ‘Disease Progression.’ Free-words included ‘upgrad*,’ downgrad*,’ ‘upstag*,’ etc. which were searched in all fields. The literature search was language-restricted to English and no article-type and publication-year restrictions were included in the search. Supplementary records were identified through reviewing references of relevant literatures.

#### Study criteria and selection

**Fig. 1:**
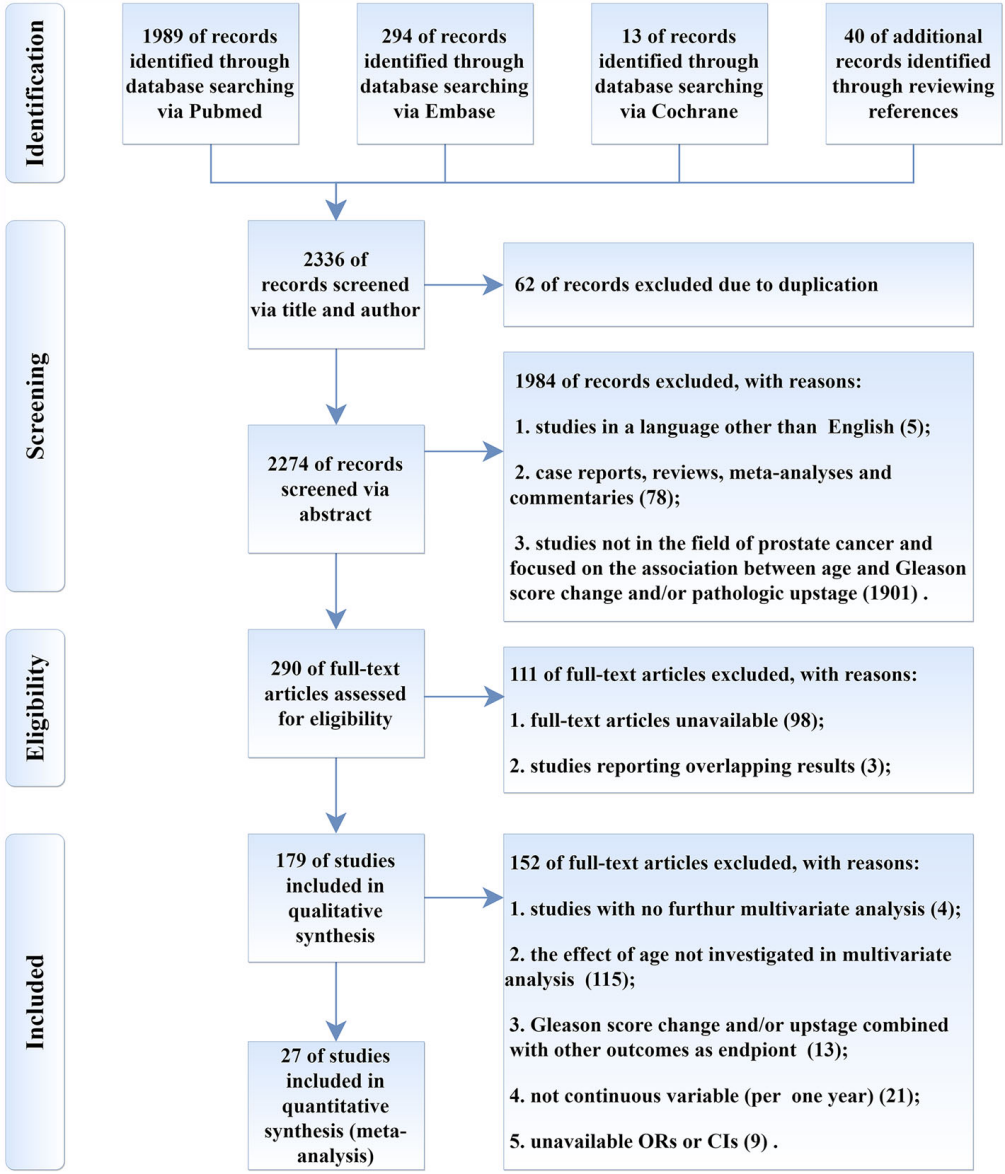
Preferred Reporting Items for Systematic Reviews and Meta-analysis (PRISMA) flow diagram

Studies were eligible if they met the following criteria: (1) original research with experimental designs; (2) studies in the research field of PCa; (3) studies focused on the association between age and GS change or upstaging; (4) statistical methods of multivariate analysis (MVA). Major exclusive criteria included (1) reviews, meta-analysis or comments; (2) studies with unavailable full-text such as conference abstract; (3) studies of sample size fewer than 50 patients. See Fig. 1 for the PRISMA flow diagram detailing the criteria.

#### Study selection

Records were retrieved from electronic databases and citations identified from relevant literatures. Obtained records were deduplicated, and the remaining were screened via titles and abstracts for full-text review. If there existed studies reporting the overlapping results (from same database and same study period), we selected the one with largest sample size. The final included literatures were evaluated in both qualitative synthesis and quantitative synthesis (meta-analysis). This whole selection procedure was conducted by two investigators (XW, YZ) independently, and disagreements were resolved by consensus and approved by a third investigator (ZJ).

#### Data extraction

Data were independently extracted by two investigators (XW, YZ) from included literatures and any discrepancies were resolved by consensus and approved by a third investigator (ZJ). Procedures of extraction were performed using a standardized form including study characteristics (first author, year of publication, region and interval of study, study size, and definition of endpoints), patient information (age, PSA, PV, GS, clinical T-stage, and number of cores obtained) and statistical data (adjusted odds ratio (aOR) with respective 95% confidence interval (CI)) (shown in Tables 1 and 2).

**Table 1:** Summary data for studies comparing outcomes between biopsy and prostatectomy included for this review

Author (year )	Study characteristic ^a^						Patient characteristic					
	Study region (interval)	Study size, *n*	No. Gleason score change, *n* (%)	No. Upstaging, *n* (%)	Definition of Gleason score change (from bGS to pGS)	Definition of upstaging	Mean age, years	Mean PSA, ng/mL	Mean PV, mL	bGS	Clinical T-stage	No. cores obtained, *n*
Audenet 2017 [14]	France (2006–2013)	1791	1093(61.0)	–	≤ 6 to ≥ 7	–	NA	NA	NA	≤ 6	T_1c–3b_	10–14
Bullock 2019 [15]	UK (2011–2016)	17598	4489(25.5)	–	Any upgrading	–	63.2	10.06	NA	> 7: 14%	T_1–4_	NR
Dinh 2015 [16]	USA (2010–2011)	10288	4467(43.4)	992(9.6)	≤ 6 to ≥ 7	cT_1–2_N_0_ to pT_3a–4_N_0–1_	59.5	5.2	NA	≤ 6	T_1c–2a_	12
Dinizo 2018 [6]	USA (2003–2014)	2256	784(34.8)	163(7.2)	≤ 6 to ≥ 7	cT_1–2_ to pT_3a–4_	NA	NA	NA	≤ 6	T_1–2a_	≥ 12
Epstein 2012 [17]	USA (2002–2010)	5071	1841(36.3)	–	≤ 6 to ≥ 7	–	NA	NA	NA	≤ 6	T_1–2a_	NR
Ferro 2019 [18]	Italy (2006–2013)	260	166(63.8)	–	≤ 6 to ≥ 7	–	62.2	5.8	51.9	> 3 + 4: 13%	T_1c–2a_	Median 10, Range 6–40
Gondo 2015 [9]	USA (2005–2013)	1317	115(8.7)	–	3 + 4 to ≤ 6	–	NA	NA	NA	3 + 4	T_1c–4_	≥ 12
Jalloh 2015 [19]	USA (1990–2012)	4231	1123(26.5)	499(11.8)	≤ 6 to ≥ 7	cT_1–2_N_0_ to pT_3a–4_N_0–1_	59.9	NA	NA	≤ 6	T_1–2_	Mean 9.15
Jeon 2017 [10]	USA and Korea (2006–2015)	854	484(56.7)	88(10.3)	≤ 6 to ≥ 7	cT_1–2_ to pT_3a–4_	NA	NA	NA	≤ 6	T_1c–2a_	NR
Leeman 2019 [11]	Germany (1992–2017)	3571	115(3.2)	245(6.9)	≤ 6 to ≥ 7	cT_1–2_ to pT_3a–4_	NA	NA	NA	≤ 6	T_1c–2_	Median 9
Luzzago 2018 [20]	Italy (2012–2016)	383	134(35.0)		bGS≤ 6, cT_1–2_N_0_ to pGS ≥ 7 and/or pT_3a–4_N_0–1_		NA	NA	NA	≤ 6	T_1c–2a_	≥ 12
Lyon 2016 [21]	USA (1999–2015)	1256	647(51.5)	–	≤ 6 to ≥ 7	–	NA	NA	NA	≤ 6	T_1–4_	≥ 6
Kim 2013 [22]	Korea (2005–2011)	451	194(43.0)	–	≤ 6 to ≥ 7	–	64	8.79	38.1	≤ 6	T_1–2_	Mean 11.5, Range 2–27
Kwon 2016 [23]	USA (2006–2015)	217	58(26.7)	–	≤ 6 to ≥ 7	–	NA	NA	NA	≤ 6	T_1–2a_	NR
Magheli 2010 [24]	Germany (1999–2004)	610	308(50.5)	–	≤ 6 to ≥ 7	–	NA	NA	NA	≤ 6	T_1–2_	NR
Martin 2017 [25]	USA (2005–2008)	136	19(14.0)	–	≤ 7 to ≥ 8	–	NA	NA	NA	≤ 7	T_1c–2_	≥ 10
Mizuno 2016 [26]	Japan (2005–2011)	284	154 (54.2)		bGS ≤ 6, cT_1–2_ to pGS ≥ 7 and/or pT_3a–4_		NA	NA	NA	≤ 6	T_1c–2a_	NR
Morlacco 2016 [5]	USA (2006–2014)	1190	156(13.1)	205(17.2)	3 + 4 to ≥ 4 + 3	cT_1–2_ to pT_3a–4_	NA	NA	NA	3 + 4	T_1c–2a_	Median 12, IQR 12–14
			201(16.9)		3 + 4 to ≤ 6							
			295 (24.8)		bGS = 3 + 4, cT_1–2_N0 to pGS ≥ 4 + 3 and/or pT_3a–4_N_0–1_							
Oh 2012 [27]	Korea (2003–2010)	505	253(50.1)	–	≤ 6 to ≥ 7	–	65.2	7.79	39.5	≤ 6	T_1c–2a_	≥ 12
Pietzak 2014 [28]	USA (1998–2008)	400	-	49(12.3)	–	cT_1-2_ to pT_3a-4_	NA	NA	NA	≤ 6	T_1c–2a_	≥ 10
Shoag 2020 [29]	USA (2010–2015)	11025	2255(45.2)	–	Any downgrading	–	NA	NA	NA	6–10	T_1–3_	NR
Weiner 2015 [30]	USA (2010–2011)	17943	8113(45.2)		bGS ≤ 6, cT_1-2_N_0_ to pGS ≥ 7 and/or pT_3a–4_N_0–1_		NA	NA	NA	≤ 6	T_1–2a_	NR
Wong 2012 [31]	Canada, Australia and UK (2003–2010)	644	333		bGS ≤ 6, cT_1–2_ to pGS ≥ 8 and/or pT_3a–4_		NA	NA	NA	≤ 6	T_1–2a_	8–12
Zanaty 2018 [12]	Canada (2006–2014)	237	134(56.5)	–	≤ 6 to ≥ 7	–	58.6	38.4	5.03	NR	T_1c–2a_	NR

*PSA* prostate-specific antigen, *PV* prostate volume, *bGS* biopsy Gleason score, *pGS* prostatectomy Gleason score, *NA* not available, *IQR* interquartile range

^a^All studies were case-control designs

**Table 2:** Summary data for studies comparing outcomes between diagnostic biopsy and confirmatory biopsy included for this review

Author (year )	Study characteristic ^a^				Patient characteristic					
	Study region (interval)	Study size, *n*	No. Upgrading, *n* (%)	Definition of upgrading (from bGS to pGS)	Mean age, years	Mean PSA, ng/mL	Mean PV, mL	bGS	Clinical T-stage	No. cores obtained, *n*
Anderson 2015 [32]	USA (1991–2001)	646	55 (8.5)	≤ 6 to ≥ 7	NA	NA	NA	≤ 6	T_1c–2a_	Median 12, IQR 11–12
Dai 2019 [33]	USA (2005–2015)	406	85 (20.9)	≤ 6 to ≥ 7	NA	NA	NA	≤ 6	T_1–2_	12
Herrera-Caceres 2020 [34]	Canada (2006–2018)	726	159 (21.9)	≤ 6 to ≥ 7	NA	NA	NA	≤ 6	NA	Median 12, IQR 10–12

*PSA* prostate-specific antigen, *PV* prostate volume, *bGS* biopsy Gleason score, *pGS* prostatectomy Gleason score, *NA* not available, *IQR* interquartile range

^a^All studies were case-control designs

In order to facilitate elucidation, studies were categorized into groups by definitions of study endpoints including (1) upgrading from biopsy to prostatectomy; (2) upgrading from diagnostic biopsy to confirmatory biopsy; (3) downgrading from biopsy to prostatectomy; (4) upstaging; and (5) upgrading and/or upstaging.

#### Risk of bias assessment

Two investigators (ZJ and QP) independently evaluated each included study utilizing the Newcastle-Ottawa Quality Assessment Scale (NOS) for case-control studies. Discrepancies in score assignment were later resolved by consensus.

#### Statistical analysis

The conversion to means of variables was roughly calculated using medians combined with interquartile range (IQR) according to Luo’s methods [35]. Random effects models were used for all meta-analysis by computing log-transformed aOR (logaOR) with respective standard error (SE) to appraise the predictive performance of age on GS change and upstaging. Forest plots were depicted to provide the pooled results (i.e., the overall effect measure, Z statistic) and heterogeneity among studies. Heterogeneity was evaluated using *I*^2^ statistic, which represented whether the variation was attributed to heterogeneity or chance. Further subgroup analysis and sensitivity analysis were carried out to find the possible sources of heterogeneity. The publication bias was evaluated by visually inspecting the asymmetry of funnel plots and subsequently quantified by Egger’s tests. Tests of publication bias were only used when there were at least 10 studies included in each group otherwise power of tests were too low to distinguish chancer from real asymmetry. Tests were 2 sided and *P* = 0.05 was the threshold for statistical significance. Meta-analysis and statistical tests were performed using computer software of RevMan version 5.3 and Stata version 12.0.

### Evidence synthesis

There were 2296 eligible records retrieved from electronic databases and additional 40 identified from pertinent references. Of retrieved records, 62 were deduplicated and the remaining 2274 were screened for full-text review via titles and abstracts. Two hundred ninety articles were selected after screening, and 27 literatures were finally included according to exclusive criteria. The reasons for exclusion were provided in PRISMA flow diagram (shown in Fig. 1).

#### Study description

Included 27 literatures were all retrospective studies of case-control designs which were published between 2010 and 2020. Eighteen literatures were monocentric studies, 4 were multicentric, and 5 were from administrative databases. Total sample size of this review was 84,296 patients in a time span of 28 years (1990–2018). Irrespective of literatures based on administrative databases, sample size was 23,211 patients. There were 18 studies from North America, 7 from Europe, 4 from Asia, and 1 from Australia. Seventeen literatures were enrolled into group of upgrading from biopsy to prostatectomy with total 48,590 patients [5, 6, 10–12, 14–19, 21–25, 27], while 3 were enrolled into group of upgrading from diagnostic biopsy to confirmatory biopsy (*n* = 1778) [32–34]. Three literatures were eligible for group of downgrading from biopsy to prostatectomy (*n* = 13532) [5, 9, 29], 7 were eligible for group of upstaging (*n* = 22790) [5, 6, 10, 11, 16, 19, 28] and 5 were eligible for group of upgrading and/or upstaging (*n* = 20444) [5, 20, 26, 30, 31] (shown in Tables 1 and 2).

From biopsy to prostatectomy, upgrading was found in 32.3% of patients with biopsy specimens, while downgrading was found in 19.0% of patients. Upgrading from diagnostic biopsy to confirmatory biopsy was found in 16.8%. Of 22,790 patients, the upstaging was found in 9.8% of organ confined diseases. Calculated with extractable data, the pooled mean age, PSA level, and PV were 62.1 years, 3.97 ng/mL, and 42.1 mL, respectively. Most patients had low-risk organ-confined PCa at diagnosis and underwent biopsy with extended schemes (shown in Tables 1 and 2)

#### Risk of bias assessment

All included literatures had low-to-moderate risk bias according to NOS scale. Three articles were rated as a total score of 5, while the others were rated as 6 or more. Studies with data source of administrative databases instead of original records resulted in bias of selection and inaccuracy in ascertainment of exposure. Studies not adjusting for potential confounders in MVA resulted in poor comparability, especially the excluded confounders very likely influencing discordance of GS and disease stage between initial diagnosis and final outcomes (shown in Table 3)

**Table 3:** Newcastle-Ottawa Quality Assessment Scale (NOS) for included case-control studies

Study	Selection				Comparability	Exposure			Total score (sum of stars)
	Is the case definition adequate?	Representativeness of the cases	Selection of Controls	Definition of Controls		Ascertainment of exposure	Same method of ascertainment for cases and controls	Non-response rate	
Anderson 2015 [32]	★	★	☆	★	★★	★	★	★	8
Audenet 2017 [14]	★	★	☆	★	★★	★	★	★	8
Bullock 2019 [15]	☆	★	☆	★	★☆	☆	★	★	5
Dai 2019 [33]	★	★	☆	★	★★	★	★	★	8
Dinh 2015 [16]	☆	★	☆	★	★★	☆	★	★	6
Dinizo 2018 [6]	★	★	☆	★	★☆	★	★	★	7
Epstein 2012 [17]	★	★	☆	★	★☆	★	★	★	7
Ferro 2019 [18]	★	★	☆	★	★★	★	★	★	8
Gondo 2015 [9]	★	★	☆	★	★★	★	★	★	7
Herrera-Caceres 2020 [34]	★	★	☆	★	★★	★	★	★	7
Jalloh 2015 [19]	★	★	☆	★	★★	★	★	★	8
Jeon 2017 [10]	★	★	☆	★	★★	★	★	★	8
Kim 2013 [22]	★	★	☆	★	★★	★	★	★	8
Kwon 2016 [23]	★	★	☆	★	★★	★	★	★	8
Leeman 2019 [11]	★	★	☆	★	★☆	★	★	★	7
Luzzago 2018 [20]	★	★	☆	★	★★	★	★	★	8
Lyon 2016 [21]	★	★	☆	★	★★	★	★	★	8
Magheli 2010 [24]	★	★	☆	★	★☆	★	★	★	7
Martin 2017 [25]	★	★	☆	★	★☆	★	★	★	7
Mizuno 2016 [26]	★	★	☆	★	★☆	★	★	☆	6
Morlacco 2016 [5]	★	★	☆	★	★☆	★	★	★	7
Oh 2012 [27]	★	★	☆	★	★★	★	★	★	8
Pietzak 2014 [28]	★	★	☆	★	★☆	★	★	★	7
Shoag 2020 [29]	☆	★	☆	★	★☆	☆	★	★	5
Weiner 2015 [30]	☆	★	☆	★	★☆	☆	★	★	5
Wong 2012 [31]	★	★	☆	★	★★	★	★	★	8
Zanaty 2018 [12]	★	★	☆	★	★★	★	★	★	8

#### Gleason score change

**Fig. 2:**
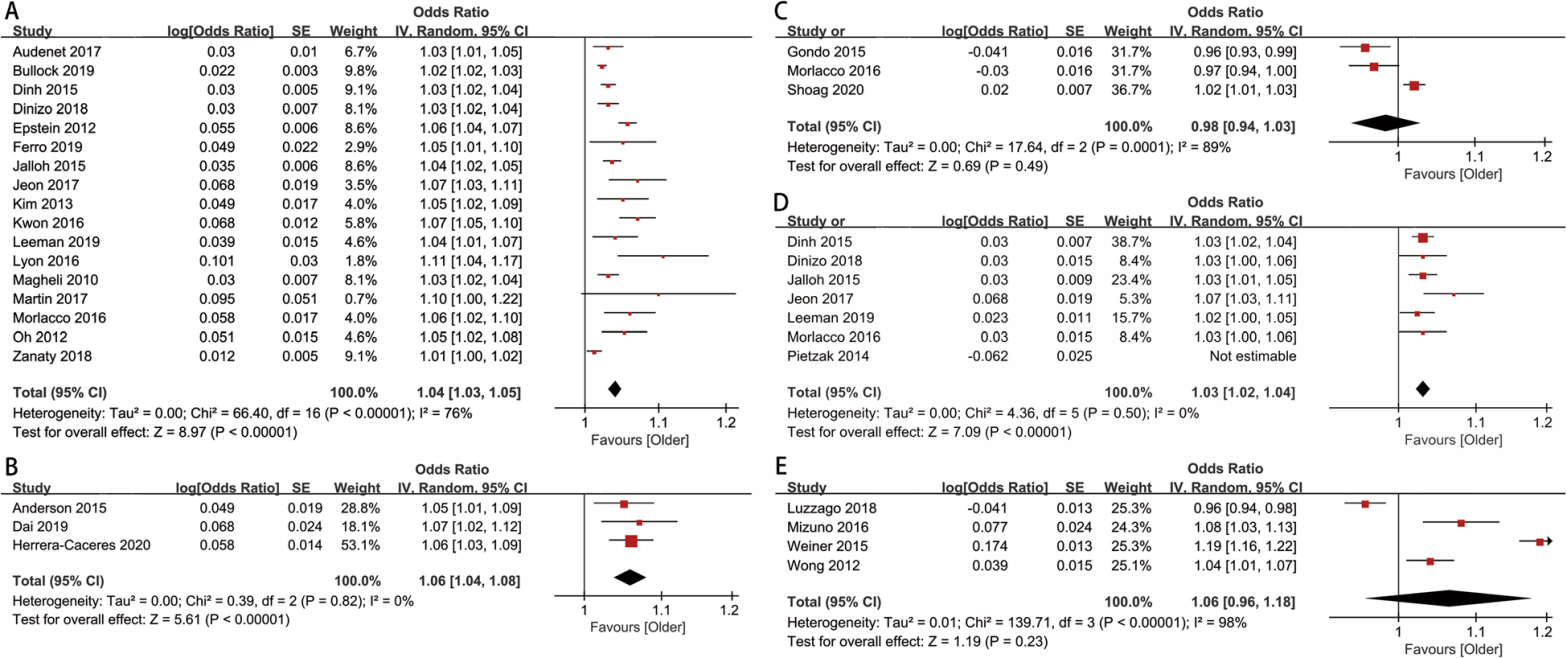
Forest plot of age predicting. **a** Group A (upgrading from biopsy to prostatectomy). **b** Group B (upgrading from diagnostic biopsy to confirmatory biopsy). **c** Group C (downgrading from biopsy to prostatectomy). **d** Group D (upstaging). **e** Group E (upgrading and/or upstaging). An odds ratio of > 1 indicates relative chance for older age versus younger age

**Fig. 3:**
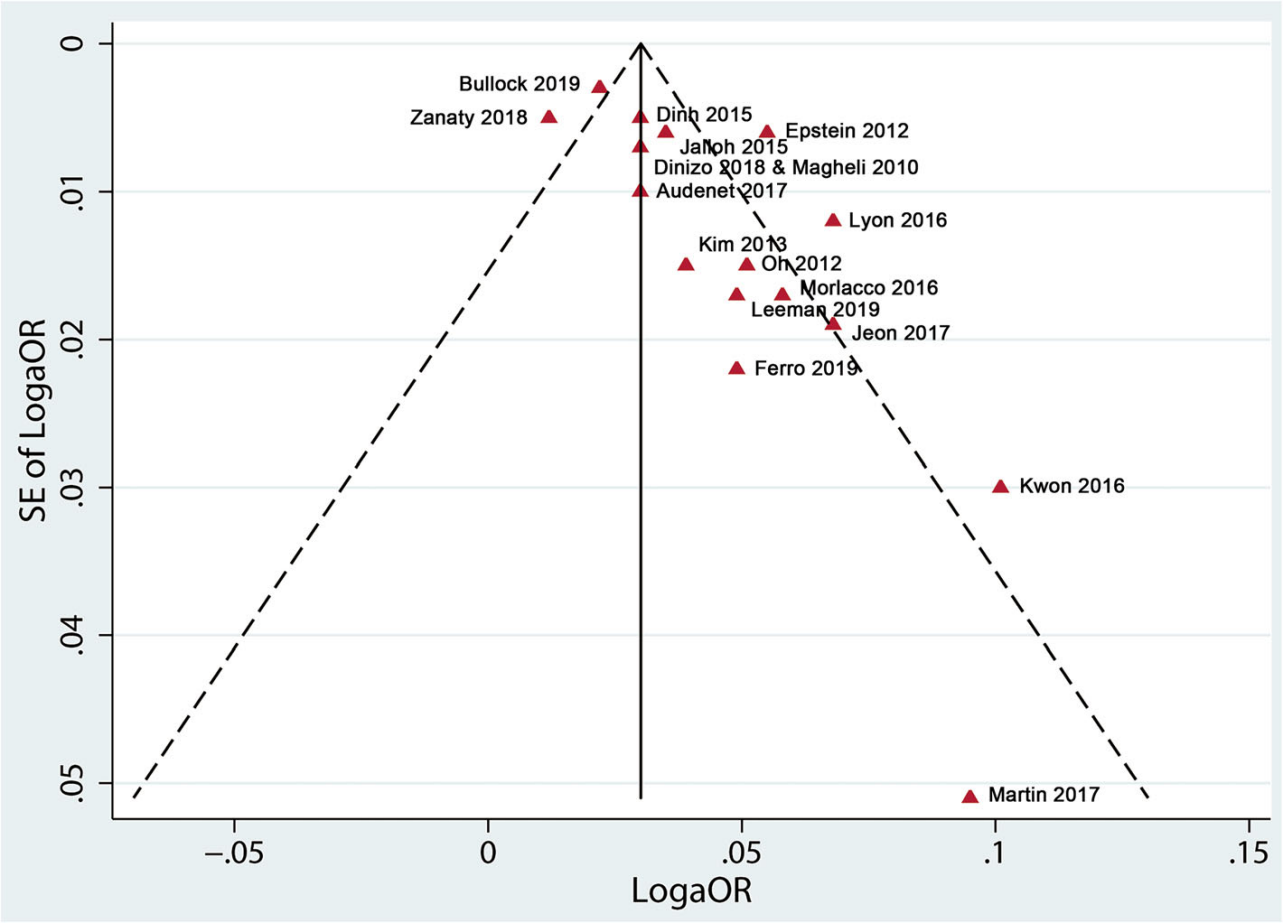
Funnel plot of studies focused on age predicting upgrading from biopsy to prostatectomy

As shown in the forest plots (shown in Fig. 2), age was found to be an independent predictor of upgrading. Older age was associated with a significant increased risk of upgrading from biopsy to prostatectomy (pooled aOR 1.04, 95% CI 1.03–1.05; *p* < 0.01; *I*^2^ = 76%). According to funnel plots and Egger’s tests (shown in Fig. 3 and Additional Figure 2), publication bias was observed in group of upgrading from biopsy to prostatectomy (*p*_Egger_ = 0.003). We found a similar direction of effect when we examined studies focused on upgrading from diagnostic biopsy to confirmatory biopsy (pooled aOR 1.06, 95% CI 1.04–1.08; *p* < 0.01; *I*^2^ = 0%). However, age failed to be associated with downgrading from biopsy to prostatectomy (pooled aOR 0.98, 95% CI 0.94–1.03; *p* = 0.49; *I*^2^ = 89%).

#### Pathologic upstaging

The pooled adjusted odds of upstaging within older men compared with the younger men were 1.03 (95% CI 1.01–1.04; *p* = 0.002; *I*^2^ = 66%). In group of studies using upgrading combined with upstaging as endpoint, no association was found between the endpoint and age (pooled aOR 1.06, 95% CI 0.96–1.18; *p* = 0.23; *I*^2^ = 98%) (shown in Fig. 2)

#### Heterogeneity analysis

In subgroup analysis and sensitivity analysis of upgrading (shown in Table 4), we managed to seek the source of heterogeneity. We found that *I*^2^ statistic decreased to 67% when studies including patients of higher than cT2 were removed. Decreased heterogeneity (62%) was also found when studies not adjusted for PV were removed. Considering small size effect, *I*^2^ statistic decreased to 44% and 12%, respectively in subgroups above after removed literatures of study size smaller than 300. After removing studies not adjusted for number of cores obtained, *I*^2^ statistic decreased to 50%. Prostate size [10, 17, 18, 21, 22], number of cores [36], and disease stage [11, 12, 15] were validated to be independent predictors of upgrading. Existence of confounders was thought to be the major reason for heterogeneity of our synthesis. As for upstaging, after removed one literature [28] of small size, *I*^2^ statistic decreased to 0%. Small size study made the pooled analysis unstable and less robust.

**Table 4:** Reasons of studies removed from meta-analysis in subgroup analysis

Study	Non-organ confined disease	Not adjusted for PV	Not adjusted for no. cores	Study size ≤ 300
Audenet 2017 [14]	**×**		**×**	
Bullock 2019 [15]	**×**	**×**	**×**	
Dinh 2015 [16]		**×**		
Dinizo 2018 [6]			**×**	
Epstein 2012 [17]	**×**	**×**	**×**	
Ferro 2019 [18]			**×**	**×**
Jalloh 2015 [19]				
Jeon 2017 [10]				
Kim 2013 [22]				
Kwon 2016 [23]		**×**	**×**	
Leeman 2019 [11]		**×**		
Lyon 2016 [21]	**×**	**×**		
Magheli 2010 [24]			**×**	
Martin 2017 [25]			**×**	**×**
Morlacco 2016 [5]				
Oh 2012 [27]		**×**		
Zanaty 2018 [12]			**×**	**×**

**×** studies removed, *PV* prostate volume

## Discussion

In this systematic review and meta-analysis of 27 studies with moderate-to-high ranking of quality, we identified that age was an independent predictor for upgrading from biopsy to prostatectomy (aOR 1.04, 95% CI 1.03–1.05) and upstaging (aOR 1.03, 95% CI 1.01–1.04) in the patient groups who were eligible for curative treatment. For every increased year of age, there was a 4% increased risk of upgrading and 3% of upstaging. From diagnostic biopsy to confirmatory biopsy, there was a 6% increased risk of upgrading (aOR 1.06, 95% CI 1.04–1.08). Based on large-scale population, this review also demonstrated that upgrading, downgrading and upstaging occurred respectively in 32.3%, 19.0% and 9.8% of patients who underwent prostatectomy. Upgrading at confirmatory biopsy was found in 16.8% of initial biopsy.

PCa is a spectrum of heterogeneous diseases, some of which are so indolent to influence overall survival especially in older patients. Therefore, precise assessment of risk stratification seems to be pivotal in disease management. It has been demonstrated that upgrading and upstaging were significantly associated with biochemical recurrence, unfavorable surgical outcomes, and cancer-specific survival [7, 37, 38]. Even if the proportion of both upgrading and upstaging is small, precise risk stratification and prediction of outcomes are requisite for individualized management.

For younger individuals especially with good health status, those with low or favorable intermediate risk PCa are recommended to undergo AS rather than immediate curative therapies [1]. Even if younger patients under AS had no significant difference in treatment-free survival compared with older counterparts [39], delayed treatment can avoid side effects such as erectile and voiding dysfunction in the young. According to the findings of this review, young patients had lower risk of upgrading and upstaging. Accurate assessment of PCa may boost confidence of urologists and patients in initiation and continuation of AS protocol. For patients with low risk of upgrading and upstaging who intend to undergo definitive treatment, salvage therapies might be unnecessary to improve prognosis. Longer waits between postoperative follow-ups might be recommended. Even if the risk is relatively low, confirmatory biopsy or rebiopsy is an option before developing and implementing PCa management programs.

For older asymptomatic individuals with other morbidities or short life expectancy, no further therapies are indicated until symptoms occurs [1]. Provided that older patients might have higher rate of upgrading and upstaging, monitoring the course of disease need to be performed in a relatively short space of time and the delivery of palliative therapies for the development of symptoms might be earlier. Older men had a significantly higher clinical stage and biopsy Gleason grade at diagnosis which was associated with poorer prognosis compared with younger men [40]. However, fewer older individuals with good quality of life might benefit from curative treatment. Thus, urologists should be reminded of high-grade PCa harbored in older patients and reconsider the treatment modalities before developing a treatment program.

Included literatures all have different research purposes that focused on different variables which might impact on upgrading or upstaging. Laboratory findings [18, 23] (such as neutrophil to lymphocyte ratios), imaging parameters [20] (such as PI-RADS score), pathological features [28] (such as number or proportion of positive biopsy cores) and other clinical factors [12] (such as years of research) were all investigated to have potential predictive performances. This might contribute to the heterogeneity of our review. Age is a basic parameter as well as a confounder which is usually adjusted for in MVA. And as such, age was validated to be an independent predictor under different circumstances.

In 2005 and 2014, amendments to the Gleason grading system by the International Society of Urological Pathology (ISUP) were recommended [41]. This has led to a closer correlation between the grade of a series of thin core biopsies and the subsequent radical prostatectomy specimen. It has been reported that Gleason score agreement improved and the proportion of biopsy cores upgraded decreased from before 2005 to after 2005 [42]. However, many studies have demonstrated that age was independent predictor even if it was adjusted for year [11, 15, 19, 20, 30]. Even so, further long-term studies should also consider the version of grading system as a confounder.

It is highly debatable whether PCa progresses during long interval from biopsy to prostatectomy [43, 44]. It has been reported that actuarial risk of grade progression during 1-year follow-up was 3% among patients with Gleason 6 or less organ-confined tumors (11% for 2 years) [45]. Even if the majority of studies did not report the intervals, we assumed that variability of time did not influence the comparison among studies. However, it is worth noticing that different intervals could be the potential source of heterogeneity.

Except the heterogeneity discussed above, our review has several limitations. First of all, there was variability in study designs among our included studies in which only a few focused on the predictive performance of age. Secondly, inevitable reasons such as unavailable full-text and non-extractable data resulted in incomplete retrieval of research. Moreover, most studies only consisted of population with low-risk PCa and focused mainly on disease reclassification from GS ≤ 6 to higher, which might not represent the reality. Last but not least, different biopsy protocols influence detection rates of low-grade or high-grade diseases [36, 46]. Different schemes of biopsy and different imaging guidance which were not reported in some studies also contribute to significant variation in outcomes.

## Conclusions

Age is an independent predictor for both Gleason score upgrading and pathological upstaging. Combined with other prognostic factors, thorough consideration of age not only prompts more accurate risk stratification but also helps providers to select optimal therapies for patients with prostate cancer. Nevertheless, further robust studies are necessitated to confirm these results in the context of effect sizes for other factors such as PSA, number of positive cores, and race.

## Supplementary Information

**Additional file 1: Figure 1** The full PubMed search strategy.

**Additional file 2: Figure 2** Plot of Egger’s test studies focused on age predicting upgrade from biopsy to prostatectomy.

## Data Availability

All data generated or analyzed during this study are included in this published article [and its supplementary information files].

## Abbreviations

PCa: Prostate cancer
AS: Active surveillance
GS: Gleason score
PSA: Prostate-specific antigen
PRISMA: Preferred Reporting Items for Systematic Reviews and Meta-analysis
MVA: Multivariate analysis
PV: Prostate volume
aOR: Adjusted odds ratio
CI: Confidence interval
NOS: Newcastle-Ottawa Quality Assessment Scale
IQR: Interquartile range
logaOR: Log-transformed aOR
SE: Standard error
