# Supplementary figures and images for: Old men with prostate cancer have higher risk of Gleason score upgrading and pathological upstaging after initial diagnosis: a systematic review and meta-analysis
Source: World J Surg Oncol. 2021 Jan 20;19:18. doi: 10.1186/s12957-021-02127-3 (PMC7818761; doi:10.1186/s12957-021-02127-3)

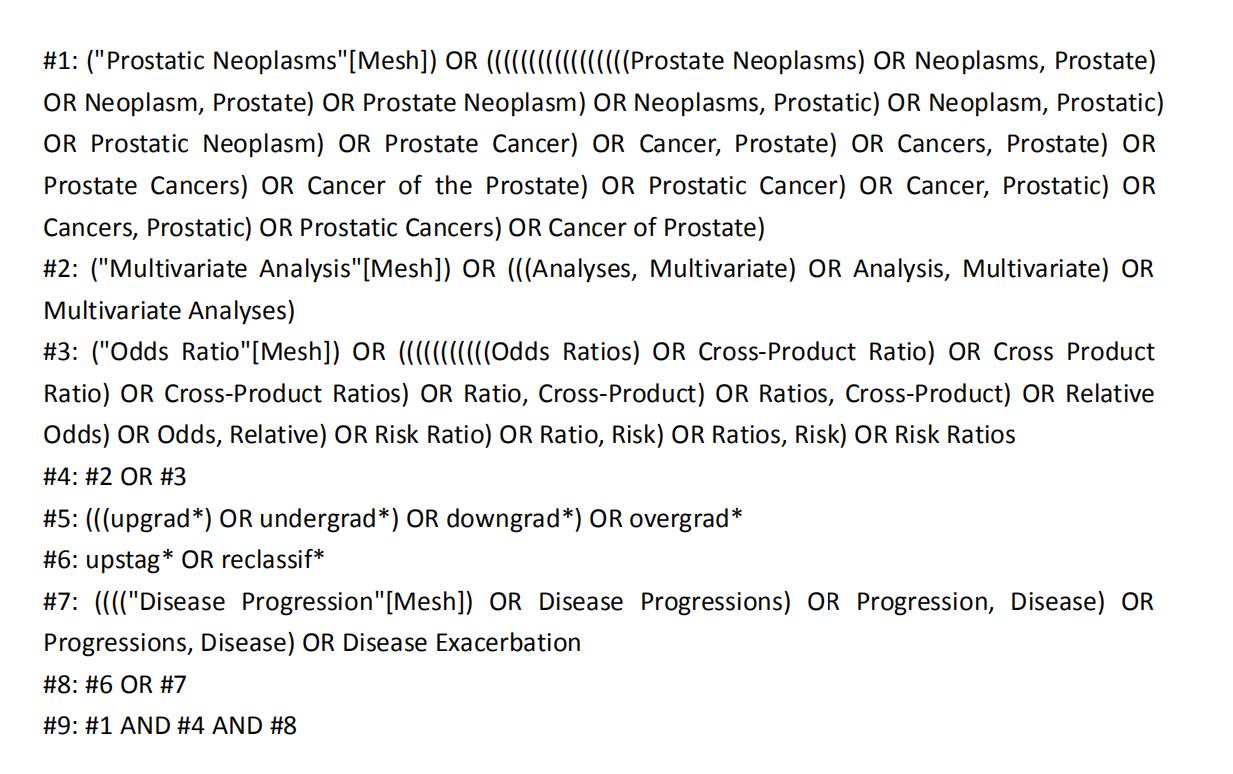

Supplement: Supplementary file 1 — Additional file 1: Figure 1 The full PubMed search strategy. [file 12957_2021_2127_MOESM1_ESM.jpg]

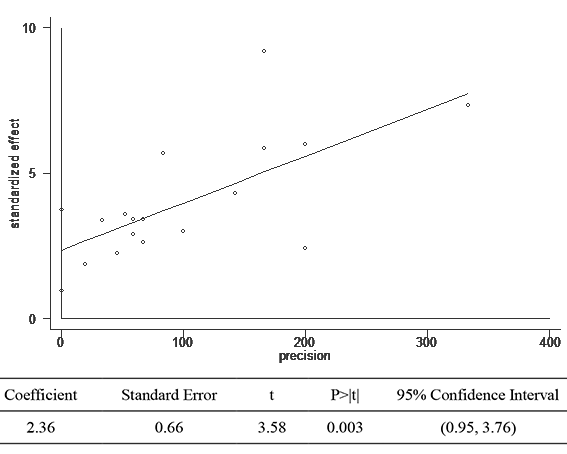

Supplement: Supplementary file 2 — Additional file 2: Figure 2 Plot of Egger’s test studies focused on age predicting upgrade from biopsy to prostatectomy. [file 12957_2021_2127_MOESM2_ESM.tif]
